# Rubia cordifolia, Fagonia cretica linn and Tinospora cordifolia exert neuroprotection by modulating the antioxidant system in rat hippocampal slices subjected to oxygen glucose deprivation

**DOI:** 10.1186/1472-6882-4-11

**Published:** 2004-08-13

**Authors:** Avinash K Rawal, Manohar G Muddeshwar, Saibal K Biswas

**Affiliations:** 1 SMV Center for Biotechnology, Sindhu Mahavidyalaya, Panchpaoli, Nagpur-440017, MS. India; 2 Department of Biochemistry, Superspeciality Hospital and Research Center, Government Medical College, Nagpur-440003, MS, India; 3 Cardiovascular Division, Biomedical Sciences, University of Edinburgh Medical School, Hugh Robson Building, George square, Edinburgh-EH8 9XE and Head, Department of Biochemistry, Dr. Ambedkar College, Deeksha Bhoomi, Nagpur-440010, MS, India

## Abstract

**Background:**

The major damaging factor during and after the ischemic/hypoxic insult is the generation of free radicals, which leads to apoptosis, necrosis and ultimately cell death. Rubia cordifolia (RC), Fagonia cretica linn (FC) and Tinospora cordifolia (TC) have been reported to contain a wide variety of antioxidants and have been in use in the eastern system of medicine for various disorders. However, their mechanism of action was largely unknown. We therefore selected these herbs for the present study to test their neuroprotective ability and the associated mechanism in rat hippocampal slices subjected to oxygen-glucose deprivation (OGD).

**Methods:**

Hippocampal Slices were subjected to OGD (oxygen glucose deprivation) and divided into 3 groups: control, OGD and OGD + drug treated. Cytosolic Cu-Zn superoxide dismutase (Cu-Zn SOD), reduced glutathione (GSH), glutathione peroxidase (GPx), nitric oxide (NO) was measured as nitrite (NO_2_) in the supernatant and protein assays were performed in the respective groups at various time intervals. EPR was used to establish the antioxidant effect of RC, FC and TC with respect to superoxide anion (O_2_^.-^), hydroxyl radicals (^. ^OH), nitric oxide (NO) radical and peroxynitrite anion (ONOO) generated from pyrogallol, menadione, DETA-NO and Sin-1 respectively. RT-PCR was performed for the three groups for GCLC, iNOS, Cu-Zn SOD and GAPDH gene expression.

**Results:**

All the three herbs were effective in elevating the GSH levels, expression of the gamma-glutamylcysteine ligase and Cu-Zn SOD genes. The herbs also exhibited strong free radical scavenging properties against reactive oxygen and nitrogen species as studied by electron paramagnetic resonance spectroscopy. In addition all the three herbs significantly diminished the expression of iNOS gene after 48 hours which plays a major role in neuronal injury during hypoxia/ischemia.

**Conclusions:**

RC, FC and TC therefore attenuate oxidative stress mediated cell injury during OGD and exert the above effects at both the cytosolic as well as at gene expression level and may be an effective therapeutic tool against ischemic brain damage.

## Background

It is generally believed that a major portion of post-traumatic neuronal necrosis after brain injury does not result from diffuse primary injury, but rather from a secondary process. The injury appears to trigger a cascade of molecular events that lead to gradual vascular and neuronal tissue degeneration, thus destroying the anatomical substrate necessary for the neurological recovery. A large body of evidence obtained from a wide variety of experimental studies of acute CNS injury strongly suggest that various reactive oxygen species (ROS) and nitrogen species (RNS) have been implicated in the progressive secondary degeneration that follows the injury [1-3]. ROS and RNS have been associated with secondary injury that amplifies the magnitude of final neuronal damage. Both biochemical analyses and studies with transgenic mice has shown that ROS/RNS production persists for many hours after the initial insult. This offers a potential therapeutic window for pharmacologic intervention of clinical relevance. Several classes of pharmacologic mimetics of superoxide dismutase/catalase have been synthesized. Evaluation of these catalytic antioxidants in laboratory models of acute brain injury has shown both robust neuroprotection and a prolonged therapeutic window at doses apparently devoid of neurotoxicity [4,5]. Another important aspect determining extent of oxidative stress mediated post-reperfusion injury subsequent to ischemia is the antioxidant status of the affected tissue as it is of great importance for the primary endogenous defence against free radical attack. Various types of antioxidant enzymes like Cu-Zn superoxide dismutase (SOD), peroxidases such as glutathione peroxidase (GPx), catalase have been reported to be neuroprotective [5,6]. Rubia cordifolia (RC), Fagonia cretica linn (FC) and Tinospora cordifolia (TC) are tropical herbs and have been extensively used in the treatment of various types of haematological, hepatic, neurological and inflammatory conditions [7]. The antioxidant and anti-inflammatory and immuno-modulatory properties of RC and TC has also been well documented [8-11]. Although RC has been reportedly used as an Ayurvedic medication in a wide variety of conditions, reports regarding the use of FC and TC are not available. In light of the aforementioned properties of RC and TC and relatively scant studies on FC we hypothesized that these herbs may overcome the oxidative stress mediated injury during ischemic neuronal injury via modulating the antioxidant pool of the cells. In order to test this hypothesis we devised a two pronged strategy in the present study to evaluate the effect of the drugs on the status of antioxidant enzymes such as GPx and Cu-Zn SOD, the levels of reduced glutathione (GSH), the principal redox regulator of the cell, and the status of nitric oxide (NO) generation the in the hippocampal slices subjected to oxygen-glucose deprivation (OGD).

## Methods

### Reagents

All reagents unless stated otherwise were obtained from Sigma Chemical Co. USA, Merck, India and Loba Chemie, India. All animals used were as per the institutional animal ethics committee approval.

### Preparation of hippocampal slices

Method for preparation of slices was similar to those of Taylor & Weber[12]. Normal New Zealand male Wistar rats weighing between 180–220 gm. were anaesthetized with ether and decapitated, whole brain was removed and placed in ice-cold oxygenated artificial cerebrospinal fluid (aCSF) containing (in mM) NaCl – 125, KCl – 3.5, CaCl_2 _– 2.0, MgSO_4 _– 1.0, NaHCO_3 _– 26, Na_2_HPO_4 _– 1.25 and D-glucose – 10 mM. The chilled brain was removed and the hippocampal regions were dissected, slices of 0.6 mm were obtained on a modified Stadie – Riggs microtome and were immersed in oxygenated aCSF between 22–28°C for 120 min to allow the tissue to get stabilized.

### Induction of in vitro oxygen-glucose deprivation (OGD) hypoxic ischemia

Hippocampal Slices were subjected to OGD (oxygen glucose deprivation) [12]. (Taylor + Weber) by suspending the slices in D-Glucose deficient aCSF equilibrated with 95% Nitrogen gas and 5% CO_2_, until the partial oxygen pressure was less then 20% as that of normoxic aCSF (pO_2 _was measured using a blood gas analyzer and was found to be ≤ 35 mm Hg). Slices were incubated in oxygen glucose deficient aCSF for 30 min at 37°C. Transferring the slices to reperfusion chamber containing aCSF started reperfusion. The extent of injury was estimated by assaying LDH release in the medium using an LDH kit.

### Study groups

Hippocampal slices were divided into 3 groups of OGD for present study.

a) The overall control group consisted of the slices immediately stabilized for 2 hours at 22–26°C in normoxic aCSF.

b) The experimental group consisted of stabilized slices subjected to OGD without any drug treatment.

c) The experimental treated group consisted of OGD slices reperfused in the medium (aCSF) containing

1) 2 mg/ml concentration of RC, TC and FC for 30 min.

2) Ascorbic acid and Reduced glutathione equivalent to their content in RC, FC and TC for 30 min.

### Biochemical assays

In all the above study groups the following biochemical parameters were monitored.

1) Free Cu-Zn superoxide dismutase (Cu-Zn SOD) was assayed using the

method of Beyer et al[13].

2) Reduced Glutathione (GSH) was assayed by the method of Teitz [14].

3) Glutathione peroxidase (GPx) was assayed by the method of Paglia et al. [15].

4) Nitric oxide (NO) was measured as nitrite (NO_2_) in the supernatant by the method of Green et al. [16].

5) Protein was estimated by the method of Lowry et al. [17].

### Electron Paramagnetic Resonance (EPR) measurement

EPR (Magnettech X-band Miniscope MS-100, Berlin Germany) was used to establish the antioxidant effect of RC, FC and TC with respect to superoxide anion (O_2_^.^), hydroxyl radicals (^.^OH), nitric oxide (NO) radical and peroxynitrite anion (ONOO) generated from pyrogallol, menadione, DETA-No and Sin-1 respectively. All the free radical donors (100 μM – 500 μM) were incubated in phosphate buffered saline (pH 7.4, 37°C) containing the spin-trap, Tempone-H (1 mM), oxidation of which generates 4-oxo-tempo with a characteristic three-line EPR signal centred at 3365 G. Development of this signal was monitored for 60 min from addition of the oxidising species and compared to parallel incubations containing RC, FC and TC (10 or 50 μM, n = 3). The amplitude of the first line of the spectrum was measured; data are expressed in arbitrary units. The EPR parameters for these experiments were as follows: Microwave frequency – 9.4 GHz; microwave power – 20 mW; modulation frequency – 100 kHz; modulation amplitude – 1500 mG; centre field 3365 G; sweep width 50 G; sweep time 20 sec; No. of passes – 1; receiver gain 3E1.

### Reverse Transcriptase Polymerase chain reaction (RT-PCR)

GCLC (Glutamyl – cysteinyl ligase catalytic subunit), iNOS (inducible nitric oxide synthase) and Cu-Zn SOD mRNA was isolated using TriZOL reagent from the hippocampal slices and reverse transcribed to study the effect of RC, FC and TC on the expression status of the above genes in OGD untreated slices after a period of 24 and 48 hours post OGD. Gene expression in treated groups was studied after 24 hours. GAPDH served as the house-keeping gene. After an initial reverse transcription, the cDNAs obtained were amplified using the following respective primers and PCR conditions:

GCLC was amplified by 32 thermal cycles of 94°C for 30 s, 55°C for 30 s, 72°C for 2 mins followed by an extension at 72°C for 10 mins using primers: *for*' 5'gtggtactgctcaccagagtgatcct and *rev*' 5'tgatccagtaactctgggcattcaca. iNOS was amplified at 94°C for 1 min, 60°C for 1 min, and 72°C for 1 min for a total of 27 cycles followed by a 10-min extension at 72°C using primers: *for*'5'-gtgttccaccaggagatgttg-3' and *rev*' 5'-tggggcagtctccattgcca-3'. Cu-Zn SOD was amplified at 94°C for 45 s, 56°C for 30 s, and 72°C for 45 s for a total of 23 cycles followed by a 10-min extension at 72°C using primers: *for*'5'-tctaagaaacatggcggtcc-3' and rev'5'-cagttagcaggccagcagat-3'. GAPDH was amplified using 20 thermal cycles of 94°C for 45 s, 60°C for 45 s, and 72°C for 1 min 30 s, followed by final extension for 10 mins at 72°C, primer used were *for'*5'ccacccatggcaaattccatggca and *rev *5'tctagacggcaggtcaggtcaacc.

## Results

### Intracellular levels of GSH is differentially modulated by RC, FC and TC

**Figure 1:**
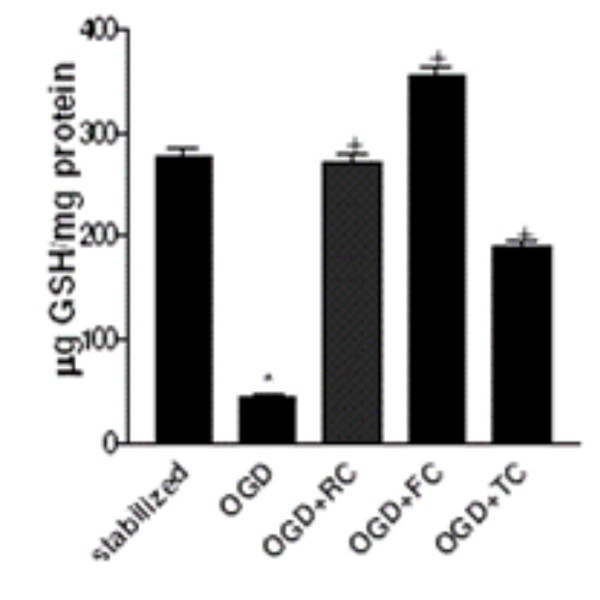
Effect of RC, FC and TC on the cytosolic GSH levels during OGD in rat hippocampal slices. * p <0.001 compared to stabilized, and + p < 0.001 compared to OGD. Results expressed are a mean of 6 independent experiments ± SEM.

Figure 1 depicts the effect of the three herbs RC, FC and TC on the intracellular GSH levels in treated rat hippocampal slices post glutamate toxicity. A significant elevation in the GSH level was recorded in untreated controls as compared to the stabilized group (p < 0.001). Where as RC and FC did show an increase in the GSH levels as compared to TC.

### Effect of RC, FC and TC on GCLC gene expression

**Figure 2:**
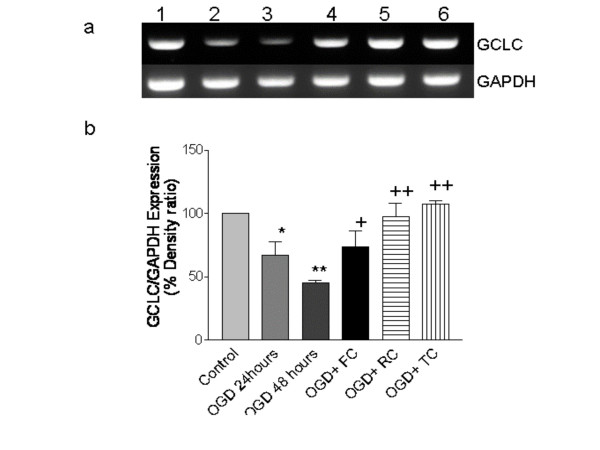
a: Representative GCLC mRNA expression in relation to GAPDH in control, OGD and OGD + drug treated rat hippocampal slices. Lane 1 – Control, Lane 2 – OGD 24 hr, Lane 3 – OGD 48 hr, Lane 4 – OGD + FC, Lane 5 – OGD+TC, Lane 6 – OGD+RC. b: Percentage densitometric expression of GCL mRNA expression in relation to GAPDH in control, OGD and OGD + drug treated rat hippocampal slices. * p < 0.01 and ** p < 0.001 versus control, + p < 0.01 versus * & ** and ++ p < 0.001 versus * & **. Results expressed are a mean of 6 independent experiments ± SEM.

Figure 2 shows the effect of RC, FC and TC on GCLC (Glutamyl – cysteinyl light chain) RT-PCR gene expression, all the three herbs were found to be inducers of the gene expression as compared to the untreated controls. Expression of housekeeping gene GAPDH was unaltered in all the lanes.

### RC, FC and TC directly scavenge free radicals

**Figure 3:**
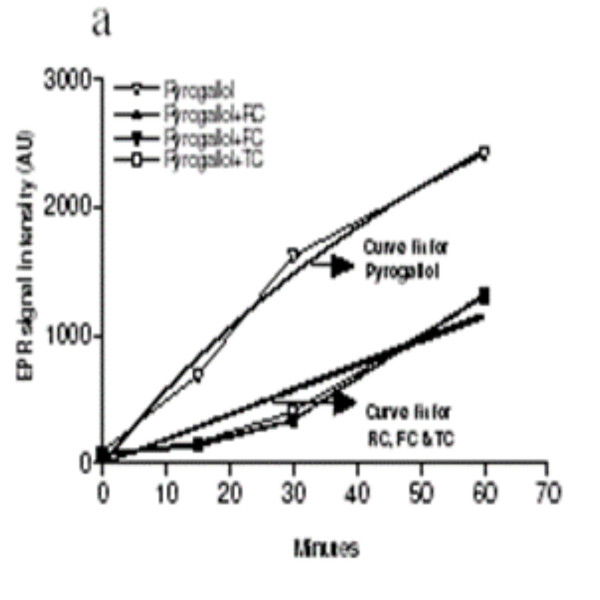
Non-linear curve fit analysis of the effect of RC, FC and TC on scavenging free radicals such as A) O_2_^-^, B) OH^.^, C) NO and D) ONOO^- ^generated by pyrogallol, menadione, DETA-NO and SIN-1 respectively. The reference curve fit is depicted in the respective groups. Results expressed are a mean of 3 independent experiments ± SEM.

RC, FC and TC contain polyphenols, a class of compound that can directly interact with electrophillic species and thus can act as a direct scavenger of free radicals. To test this hypothesis we employed EPR spectroscopy to study the interaction of RC, FC and TC with certain free radicals such as O_2_^.-^, ^.^OH, NO and ONOO using specific donors. EPR spectroscopy revealed that addition of RC, FC or TC (10 μg/ml) to the O_2_^.- ^generator, pyrogallol (100 μM; n = 3, Fig 3a) or the ^. ^OH generator, menadione (500 μM; n = 3, Fig 3b), NO generator, DETA-NO (100 μM; n = 3, Fig 3c) and ONOO generator Sin-1 (100 μM; n = 3, Fig 3d) in the absence of a spin trap failed to generate a spin signal over a 60 min period. This indicated, either the herbs interacted with the free radicals covalently and therefore yielded no paramagnetic signals or did not interact at all. However, 3-line spin signals characteristic of formation of the stable radical 4-oxo-tempo developed in a time-dependent manner when the same oxidant generating substances were incubated with the recognised spin trap, tempone-H (1 mM). Co-incubation of RC, FC and TC with pyrogallol (100 μM) in the presence of tempone-H caused a significant inhibition of spin signal development over a 60 min time period (p < 0.001, 2-way ANOVA – results of Bonferroni post-hoc analyses and non-linear curve fits are shown on the figure; n = 3).

### Intracellular levels of GPx are increased by RC, FC and TC

**Figure 4:**
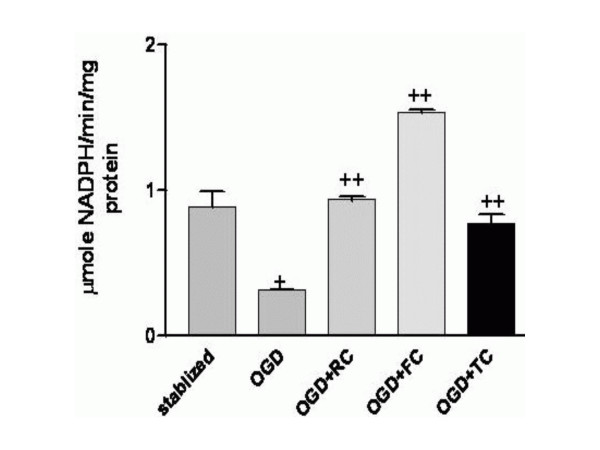
Effect of RC, FC and TC on the glutathione peroxidase levels in rat hippocampal slices. + p < 0.01 versus stabilized and ++ p < 0.001 versus OGD. Results expressed are a mean of 4 independent experiments ± SEM.

Figure 4 depicts the effect of the three herbs RC, FC and TC on the intracellular GPx levels in treated rat hippocampal slices post glutamate toxicity. A significant rise in the GPx level was recorded in treated slices as compared to the stabilized and control group (p < 0.001). Here too RC and FC show an increase in the GPx levels as compared to TC.

### Cu-Zn SOD gene expression and enzyme level is upregulated by RC, FC and TC

**Figure 5:**
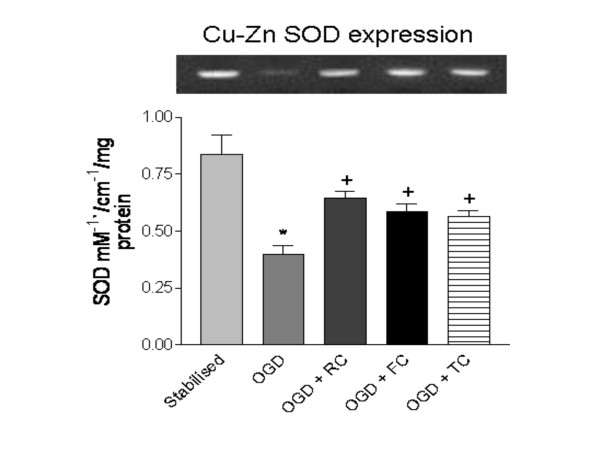
Effect of RC, FC and TC on Cu-Zn SOD gene expression and cytosolic free levels during OGD in rat hippocampal slices. RT-PCR pictograph of Cu-Zn SOD is shown aligned to the corresponding groups. * p < 0.001 versus stabilized, + p < 0.001 versus OGD. Results expressed are a mean of 6 independent experiments ± SEM.

Figure 5 depicts the effect of the three herbs RC, FC and TC on the expression and activity levels of intracellular Cu-Zn SOD in stabilised, untreated and treated rat hippocampal slices post OGD. A significant drop in the SOD level was recorded in untreated OGD slices as compared to the stabilized group (p < 0.001). A significant rise in the levels of both SOD gene expression and the enzyme activity was observed for RC, FC and TC (p < 0.001).

### RC, FC and TC and exogenous antioxidants decrease nitric oxide generation and iNOS gene expression

**Figure 6:**
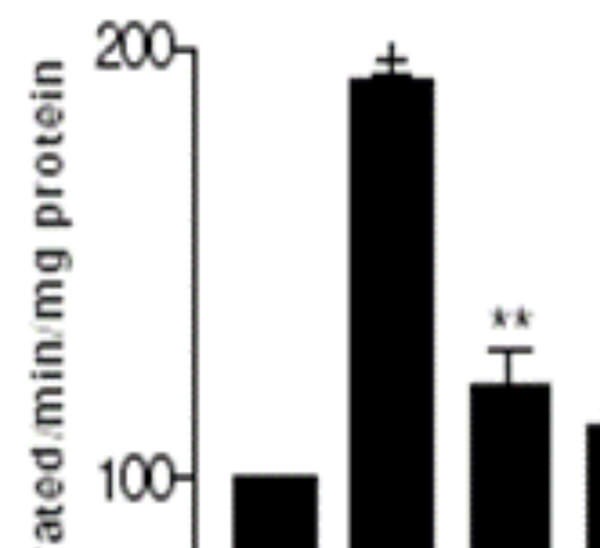
Effect of RC, FC and TC and amounts of ascorbic acid and GSH equivalent to that present in the herbs (RC*, FC* and TC* respectively) on NO_2 _generation in hippocampal slices. + p <0.001 versus control (100%) and ** p < 0.001 versus OGD. Results expressed are a mean of 3 independent experiments ± SEM.

**Figure 7:**
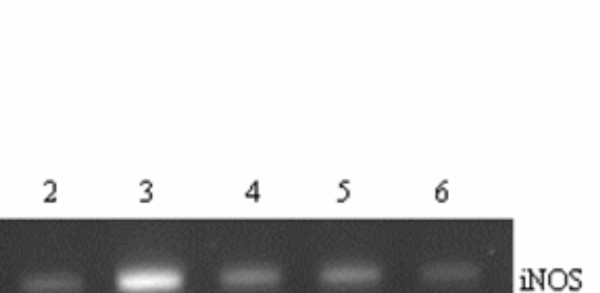
Effect of RC, FC and TC on the iNOS gene expression in rat hippocampal slices subjected to OGD and OGD+RC/FC/TC. Lane 1 – Control, Lane 2 – OGD 24 hr, Lane 3 – OGD 48 hr, Lane 4 – OGD + FC, Lane 5 – OGD+TC, Lane 6 – OGD+RC. *p < 0.05 vs control, **p < 0.001 vs control, +p < 0.001 vs **. Results expressed are a mean of 6 independent experiments ± SEM.

The effect of exogenously added antioxidants namely ascorbic acid and reduced glutathione equivalent to their concentrations found in three herbs RC, FC and TC on nitric oxide generation in comparison to the three herb extracts is shown in figure 6. All the three herbs along with the exogenously added GSH and Vit C significantly inhibited NO_2 _generation in the treated OGD slices. The decrease in the level of NO_2 _was found to parallel a decrease observed for the expression of the iNOS gene in the same group of the hippocampal slices (Figure 7).

## Discussion

Ischemic cells are known to be under oxidative stress and hence experience oxidative injuries like membrane alterations, lipid peroxidation, increased ionic influx, especially Ca and Na etc. [18-20] elevated levels of O_2_^-^, NO, NO_2_, decreased activity of Ca^2+ ^Mg^2+^ATPase and Na^+^/K^+ ^ATPase [21]. In the present study we have reported the protective effects of RC, FC and TC during OGD insult to rat hippocampal slices. GSH was protected from depletion during the insult and this protection could be correlated to an elevation in the expression of the g-GCS gene. The peroxide scavenging enzyme, glutathione peroxidase (GPx) activity was also significantly restored by the three plant extracts. Treatment of the OGD-hippocampal slices with pure vitamin C and GSH in proportions equivalent to that found in the extracts exhibited a parallel effect on NO generation as to that found with the drugs. RC, FC and TC effectively reduced free radical levels by mechanisms involving increased expression of Cu-Zn SOD, decreased expression of iNOS and simultaneous scavenging of the free radicals such as O_2_^-^, OH^.^, NO and ONOO. Overall, RC, FC and TC exhibit potential cytoprotective ability in rat hippocampal slices subjected to OGD.

In addition to its role as an antioxidant, the GSH status of a cell is critical for various other biological events that include transcriptional activation of specific genes and modulation of redox-sensitive signal transduction and hence pro-inflammatory processes during cerebral ischemia [22]. GSH also plays a crucial role in the regulation of expression of several redox-sensitive antioxidant and anti-inflammatory genes [23], processes which are aggravated especially, post-ischemic insult as a result of reperfusion of white blood cells to the injured area [24]. As a result there is a rapid loss of reducing equivalents of the cell and hence an onset of oxidative stress. The oxidative stress further leads to the upregulation of expression of a wide variety of pro-inflammatory cytokines, including adhesion molecules, all of which contribute to tissue injury, apoptosis/necrosis [25,26]. Therefore maintenance of GSH pool and other antioxidant levels is critical to cell survival and adaptation to the ischemic injury [27]. In response to the battery of free radicals generated during ischemia, the cells initially neutralize the oxidative challenge via GSH mediated antioxidant mechanisms. However, a rapid decline in the levels of GSH soon follows which ultimately leads to tissue injury. Therefore it is imperative that any therapeutic intervention should be able to cater to this deficiency observed during ischemia/OGD. Our results show that RC, FC and TC were able to reverse the GSH levels, which was significantly depleted during OGD. This restorative/protective activity of RC, FC and TC could be partly attributed to their native antioxidant contents (GSH = 8.33 ± 0.5, 10.26 ± 0.55 and 6.94 ± 0.49, Vit C = 27.52 ± 0.93, 32.99 ± 1.03 and 41.86 ± 0.68 and Polyphenols = 18.33 ± 2.02, 11.88 ± 1.33 and 21.00 ± 1.26 mg/g extract respectively). However, it was not clear at this juncture as to how much of these antioxidants are actually bio-available, an area which is out of scope of the present study. On the other hand RC, FC and TC may exert such a restorative effect by increasing the synthesis of GSH in the cells. To test this hypothesis we studied the expression status of γ-GCS gene during OGD in both untreated and treated hippocampal slices. RC, FC and TC showed a positive inductive effect on the γ-GCS gene expression after 48 hours. Although significant, it is to be noted that the three drugs exhibited partial differences in their response towards the expression of the GCLC gene expression and in restoration of the GSH levels. This indicates that the drugs may act via other mechanisms that may have a sparing effect on the GSH levels. Scavenging of the free radicals is one such mechanism whereby depletion of GSH is prevented [28,29]. In order to test the later hypothesis we examined whether or not RC, FC and TC could directly interact with the free radicals such as O_2_^-^, OH^.^, NO and ONOO using pyrogallol, menadione, DETA-NO and Sin-1 as respective donors. Electron paramagnetic resonance study has revealed a significant scavenging effect of the three drugs on the free radicals chosen for the study. The results were more pronounced for OH^. ^and NO radicals as depicted by the non-linear curve fit analysis. The effects on O_2_^-^, and ONOO radicals were also found to be quite promising. Therefore, direct scavenging of the free radicals is an important mechanism by which the drugs may exert their cytoprotective effect, not only by sparing GSH utilization by free radicals but also preventing the free radical mediated tissue damage.

GSH depletion can also be brought about by its abnormal redirection towards neutralisation of the oxidants. Within the normal metabolic course of the cell, reduction of the prostanoid hydroperoxides to their respective hydroxides, is catalysed by the enzyme glutathione peroxidase (GPx), which requires GSH as one of the co-substrates for the reaction [30,31]. This is basically a protective mechanism of the cell against the harmful lipid peroxides generated during their synthesis and oxidative stress. In the present study we have recorded a significantly decreased GPx activity in OGD hippocampal slices as compared to the controls. The observed decrement may be attributed to the depleted GSH levels either due to increased utilisation and/or diminished activity of γ-GCS. Furthermore, diminished GPx activity, at least in part, indicates cellular accumulation of the lipid hydro peroxides, which can potentially turn on a chain reaction wherein more unsaturated lipids become targets for further peroxidative tissue injury. The ability of RC, FC and TC to enhance the GPx activity is therefore an important finding since one of the protective mechanisms of the herbs under study might be mediated via upregulation of the GPx activity. This effect may further be explained in view of the fact that the herbs themselves contain an appreciable amount of GSH and ascorbate.

A large body of evidence suggests that another important intracellular enzyme Cu-Zn SOD is found to be neuroprotective in nature along with GPx and GSH [32,33]. We observed a significant drop in the cytosolic free levels of Cu-Zn SOD in the untreated OGD slices as compared to the stabilized and the three herbs RC, FC and TC significantly restored the levels of the antioxidant enzyme. However, it was not clear at this juncture as to the mechanism involved in such a restoration. Oxidative stress is known to induce the synthesis of Cu-Zn SOD as a defensive mechanism aimed at containing the oxidant levels generated during inflammatory/ ischemic conditions [34]. Cu-Zn SOD is one of the first lines of defense against free radicals such as O_2_^- ^and NO and acts as a direct scavenger of these extremely potent hazardous species. We therefore investigated whether the antioxidant properties exhibited by the three herbs also involve their modulatory effect on the expression of the Cu-Zn SOD expression. The increased expression of the Cu-Zn SOD gene in RC, FC and TC treated OGD slices clearly confirm the positive modulatory effect the three drugs have on the cellular antioxidant system. Increased expression of Cu-Zn SOD in response to these herbs is a crucial finding especially since this enzyme is directly implicated in the scavenging of not only O_2_^- ^but also the more potent toxicant ONOO [35]. Thus the three herbs show potent antioxidant properties via modulating the expression of the antioxidant genes such as GCLC and Cu-Zn SOD.

The mechanism of antioxidant and neuroprotective effects reported above for RC, FC and TC are new findings. However, as to what components of the drugs bring about such cell-protective effects could not be fully ascertained. Preliminary analysis has revealed that all the three herbs have significant amounts of GSH and Vit C and another important antioxidant, polyphenol. In addition inductively coupled plasma spectroscopic analysis have also revealed the presence of important trace elements in the three herbs (personal observations (Zn- 13.81, 18.19 and 14.94, Cu- 3.16, 9.46 and 13.79, Vd- 30.00, 18.55 and 26.00, Se- 1.79, 3.33 and 0.28 and Mo- 0.39, 1.64 and 1.15 ppm in RC, FC and TC respectively). We therefore thought that the three herbs exert their ameliorative properties due to their antioxidant and trace element contents. In order to test this hypothesis we investigated the effect of GSH and Vit C on NO generation in OGD treated and untreated hippocampal slices. The amount of GSH and Vit C used were equivalent to that found in the three herbs. Our results indicate that GSH and the Vit C components of the herbs are at least partly responsible for the attenuation of NO generation in the treated OGD slices. The effects of RC, FC and TC were almost similar to those recorded for GSH and Vit C. NO is an important neurotransmitter in the brain and also can be rendered harmful due to unprecedented generation during hypoxic/ischemic/inflammatory conditions [20,36]. In addition, NO can combine with O_2_^- ^to form a more toxic ONOO, which is extremely deleterious to the cells [37]. Hence our observation of the ability of the herbs to attenuate NO generation is not only new but also is indicative of the possible mechanism by which these herbs might exert their protective functions. It is interesting to note that RC, FC and TC also increase Cu-Zn SOD expression thus contributing to the decrease in ONOO formation due to an increased scavenging of O_2_^-^. Furthermore the three herbs were found to repress the expression of the iNOS gene, which is considered to be an important damaging factor during hypoxia/ischemia [37]. Thus the decrease in generation of NO by RC, FC and TC is not only due to direct scavenging by the herbs (fig 3) but also via the transcriptional modulation of the iNOS gene, which is induced during OGD.

## Conclusions

In conclusion, RC, FC and TC exert cell/neuroprotective properties via preventing the depletion and increasing GSH levels by inducing GCLC expression, by reducing oxidant levels via direct scavenging, decreasing iNOS expression and by increasing the antioxidant gene Cu-Zn SOD. Further protective ability may be attributed to the enhanced activity of GPx brought about by the herbs. The antioxidant contents of the herbs appear to be important components for the observed effects. However, further investigations are required to ascertain the role of individual constituents in the efficacy of the above described properties of the herbs in order to ascribe potential pharmacological applications to these herbs.

## Competing interests

Authors do not have any competing interest with anyone whatsoever.

## Authors' contributions

AKR – This work is a part of AKR's PhD thesis and he has done all the experiments, data collection and processing and manuscript preparation.

MGM – He is co-supervisor for AKR and was involved in the ideology, manuscript preparation and providing facilities for the work.

SKB – He is the supervisor for AKR and was involved in the main ideology and study design of the work, manuscript preparation and performing EPR studies and training AKR for the respective techniques.

## Pre-publication history

The pre-publication history for this paper can be accessed here:


